# Downregulation of microRNA-512-3p enhances the viability and suppresses the apoptosis of vascular endothelial cells, alleviates autophagy and endoplasmic reticulum stress as well as represses atherosclerotic lesions in atherosclerosis by adjusting spliced/unspliced ratio of X-box binding protein 1 (XBP-1S/XBP-1U)

**DOI:** 10.1080/21655979.2021.2006862

**Published:** 2021-12-16

**Authors:** Peipei Ge, Mingxiao Gao, Juan Du, Jingbin Yu, Lei Zhang

**Affiliations:** a Department of Cardiology, Yantai Yuhuangding Hospital Affiliated to Qingdao University, Yantai, People’s Republic of China; b Department of Cardiology, Rizhao Hospital of Traditional Chinese Medicine, Rizhao, People’s Republic of China; c Department of Cardiology, Zibo Central Hospital, Zibo, People’s Republic of China

**Keywords:** Atherosclerosis, miR-512-3p, autophagy, endoplasmic reticulum stress, XBP-1

## Abstract

AS is an important pathological basis of cardiovascular disease. miRNAs are involved in almost all steps of AS, including the injury and dysfunction of endothelial cells and vascular smooth muscle cells. This work elucidated the biological functions of miR-512-3p in AS and probed into the underlying molecular mechanism. In the present work, ox-LDL-treated HUVECs served as the in vitro model of AS and ApoE-/- mice were nourished with a high-fat diet to establish an in vivo model of AS. Proliferation, apoptosis, and migration of HUVECs were evaluated by CCK-8, TUNEL staining, Western blot, and transwell assays. Immunofluorescence examined LC3 expression and levels of autophagy-related and ER stress-related proteins were determined by Western blot assay. In addition, starBase predicted the complementary binding sites of XBP-1 to miR-512-3p and luciferase reporter assay confirmed the interaction between miR-512-3p and XBP-1. Moreover, H&E staining was employed to evaluate atherosclerotic lesions in AS model mice. Results revealed that ox-LDL treatment decreased the proliferative and migrative activities and promoted the apoptosis of HUVECs as well as induced autophagy and ER stress, which were abrogated by miR-512-3p silencing. Importantly, ox-LDL treatment elevated miR-512-3p expression and XBP-1 was a direct target of miR-512-3p. Mechanistically, knockdown of miR-512-3p enhanced the viability, suppressed the apoptosis, and promoted the migration of ox-LDL-treated HUVECs, alleviated atherosclerotic lesions in AS model mice as well as repressed autophagy and ER stress by targeting XBP-1 to manipulate the ratio of XBP-1S/XBP-1 U.

## Introduction

Atherosclerosis (AS) is a common vascular disease that affects the arteries from the intima. AS is the main pathobiologic basis of coronary heart disease, cerebral infarction, peripheral vascular disease, and so on, seriously threating human health [1,2]. Despite the fact that the cognitive mechanisms of AS and therapies for atherosclerotic vascular diseases have been substantially improved, the mortality rate and economic burden remain high till now [3,4]. Therefore, it is of great necessity to strengthen the prevention and treatment of AS.

As a vital element in the vascular wall, vascular endothelial cells secrete multivariate cytokines and intercellular substances to maintain vascular system stability and to prevent vascular diseases [5,6]. Autophagy, a lysosomal-dependent protein metabolic balance process, participates in the maintenance of stable internal environment [7]. Importantly, the autophagy level of vascular endothelial cells is tightly related to the pathogenesis and development of AS [8].

MiRNA is a small single stranded RNA with a length of about 22 nucleotides. It can regulate gene expression at the posttranscription level and play an important role in human growth, development, physiology, and pathology [9]. Numerous researches have reported that miRNAs are involved in almost all steps of AS, including the injury and dysfunction of endothelial cells and vascular smooth muscle cells [10,11]. Liu et al [12] proved that miR-130a was up-regulated in AS mice, and miR-130a knockdown suppressed the inflammation of LPS-induced human umbilical vein endothelial cells (HUVECs) in an in vitro model of AS. Tian et al [13] indicated that miR-378 c could regulate two key elements (VSMC phenotypic modulation and the formation of foam cells) of AS. In addition, it was verified that miR-512-3p was expressed at high levels in thrombospondin-1-induced vascular smooth muscle cells [14]. However, the biological functions of miR-512-3p in AS have not yet been elaborated.

Endoplasmic reticulum (ER) is a key organelle that can regulate protein folding, transport, and post-transcriptional modification. Stimulation from various internal and external environments causes the accumulation of misfolded or unfolded proteins in ER, ultimately resulting in ER stress [15,16]. Severe and persistent ER stress activates the unfolded protein response (UPR), which eventually leads to apoptosis and diseases [17]. A lot of literature has confirmed that ER stress plays a vital role in the pathogenesis of various cardiovascular diseases, including AS [18,19].

Furthermore, the downstream target genes of miR-512-3p is predicted using starbase database. X-box binding protein-1 (XBP-1) is one of the potential targets of miR-512-3p, revealing the relationship between miR-512-3p and ER stress. As a key regulator of UPR, XBP-1 can be divided into the active isoform (XBP-1S) and the inactive isoform (XBP-1 U). The generation of XBP-1S induces the UPR response while XBP-1 U is independent of UPR activation [20,21].

Herein, ox-LDL-treated HUVECs served as the in vitro model of AS and ApoE-/- mice were nourished with a high-fat diet to establish an in vivo model of AS. Proliferation, apoptosis, and migration of HUVECs, autophagy, ER stress as well as atherosclerotic lesions were evaluated, so as to determine the biological functions of miR-512-3p in AS and explore the molecular mechanism underlying the participation of miR-512-3p in the progression of AS.

## Materials and methods

### Cell culture

HUVECs were purchased from American Type Culture Collection (ATCC, VA, USA) and cultured in RPMI-1640 medium (Gibco, MD, USA) supplemented with 10% fetal bovine serum (FBS; Gibco, MD, USA) and 100 U/mL penicillin/100 μg/mL streptomycin (Gibco, MD, USA) in a humidified incubator at 37°C with 5% CO_2_.

### Cell treatment

HUVECs were treated with different concentrations of ox-LDL (1, 10, 100, 500 µg/mL; Solarbio, Beijing, China).

### Cell transfection

For knockdown of miR-512-3p, the miR-512-3p inhibitor and its negative control (inhibitor NC) were obtained from GenePharma (Shanghai, China). In brief, cells (4 × 10^4^ cells per well) were seeded in 12-well plates and cultured overnight. Transfection of miR-512-3p inhibitor and inhibitor NC was performed using Lipofectamine 2000 (Invitrogen, CA, USA). Cells were harvested for further analysis after 48 h transfection.

### Establishment of AS animal model

ApoE-/- male mice (8 weeks old) and C57BL/6 N (WT) male mice were purchased from the Beijing Laboratory Animal Center, Chinese Academy of Science. ApoE-/- mice were nourished with a high-fat diet (15% fat, 0.25% cholesterol) to establish an in vivo model of AS. C57BL/6 N (WT) mice were fed on a high-fat diet as the control. miR-512-3p antagomir or antagomir NC (GenePharm, Shanghai, China) were injected into ApoE-/- mice through the tail vein for 10 days, once a day. Mice were sacrificed after 12 weeks for the later experiments. The animal experiment of this study was endorsed by the Animal Research Ethics Committee of Yantai Yuhuangding Hospital Affiliated to Qingdao University.

### Cell counting kit-8 (CCK-8) assay

CCK-8 assay was employed to detect the viability of HUVECs. HUVECs were seeded into 96-well plates at a density of 5000 cells/well. 24 h post incubation, 10 µl CCK-8 reagent (Beyotime, Shanghai, China) was added into each well for additional 2 h incubation. The absorbance at 450 nm was measured with a microplate reader (Bio-Tek, GA, USA).

### TUNEL-staining assay

Apoptosis of HUVECs was assessed by performing TUNEL staining. Cells were fixed with paraformaldehyde for 30 min. After washing with PBS, cells were permeabilized with 0.3% Triton-X 100 for 5 min. Then, cells were incubated with TUNEL reaction buffer (Beyotime, Shanghai, China) for 1 h at 37°C in the dark. DAPI was used to stain the nucleus of apoptotic cells for 5 min at room temperature. The images of TUNEL-positive cells were captured by a fluorescence microscope (Leica, Wetzlar, Germany).

### Transwell assay

For the migration assays, HUVECs (2 × 104) were re-suspended in serum-free medium and seeded into the upper chamber of transwell chambers (Corning, NY, USA). A total of 600 µl medium containing 20% FBS was added into the lower chambers. After 24 h incubation, cells were fixed with 4% paraformaldehyde for 10 min and stained with 1% crystal violet solution for 20 min. Images were photographed under an inverted light microscope (Olympus, Tokyo, Japan).

### Immunofluorescence

Cells were fixed with 4% paraformaldehyde for 15 min at 4°C. Then, cells were permeabilized with 0.25% Triton X-100 for 10 min. After sealing by 1% BSA blocking solution for 1 h, cells were incubated with anti-LC3 antibody overnight at 4°C. On the second day, cells were incubated with secondary antibody for 1 h at room temperature, and the nucleus of cells was stained using DAPI. Images were observed under a fluorescence microscope (Leica, Wetzlar, Germany).

### Western blot assay

HUVECs were lyzed using RIPA lysis buffer (Beyotime, Shanghai, China) and cell supernatants were collected by centrifugation. The protein concentration was determined using BCA Protein Assay Kit (Beyotime, Shanghai, China). Protein samples were isolated by SDS-PAGE and transferred to PVDF membranes. After blocking with 5% BSA, membranes were incubated with primary antibodies against Bcl-2 (Abcam, ab32124, 1:1000), Bax (Abcam, ab243140, 1:500), Cleaved caspase-3 (Abcam, ab2302, 1:500), Caspase-3 (Abcam, ab184787, 1:2000), Beclin-1 (Abcam, ab207612, 1:2000), Atg5 (Abcam, ab108327, 1:10,000), p62 (Abcam, ab207305, 1:1000), GRP78 (Abcam, ab108615, 1:10,000), CHOP (Abcam, ab11419, 1:1000), Caspase-12 (Abcam, ab62484, 1:2000), XBP-1 (Abcam, ab220783, 1:1000) and GAPDH (Abcam, ab32124, 1:1000) overnight at 4°C. Membranes were washed in TBST, followed by incubation with secondary antibodies (Abcam, ab205718, 1:50,000; Abcam, ab205719, 1:20,000) for 1 h at room temperature. Protein bots were visualized using an enhanced chemiluminescence (ECL) detection kit (Beyotime, Shanghai, China).

### Reverse transcription-quantitative polymerase chain reaction (RT-qPCR)

Total RNA was extracted from HUVECs using TRIzol reagents (Takara, Tokyo, Japan). Then, RNA was reversely transcribed into cDNA with PrimeScript™ RT Reagent Kit (Takara, Tokyo, Japan). RT-qPCR was processed on the Applied Biosystems 7900 Real-Time PCR System using a SYBR green PCR reagent kit. The PCR conditions were as follows: 94 °C for 10 min, followed by 42 cycles of 94 °C for 15 sec and 60 °C for 60 sec. The sequences of the primers were as follows: miR-512-3p forward: 5′- AAGUGCUGUCAUAGCUGAGGUC −3′, reverse: 5′- UUCUCCGAACGUGUCACGUTT −3′; U6 forward: 5′- CTCGCTTCGGCAGCACA −3′, reverse: 5′- AACGCTTCACGAATTTGCGT −3′. U6 served as the internal control. The relative gene expression was calculated using 2^−ΔΔCt^ method.

### Hematoxylin and eosin (H&E) staining

The aorta vessel tissues were fixed in 4% paraformaldehyde. Then, after dehydration, tissues were embedded in paraffin and cut into 4 µm sections. After that, tissues were stained with H&E in line with a standard protocol and observed under an optical microscope (Leica, Wetzlar, Germany).

### Dual-luciferase reporter assay

XBP-1 3′UTR fragments containing the WT/MUT (wild type/mutated) binding sites of miR-512-3p were synthesized and cloned into pmirGLO reporter plasmid (Promega, WI, USA) to generate XBP-1 3′UTR-WT and XBP-1 3′UTR-MUT. Then, XBP-1 3′UTR-WT and XBP-1 3′UTR-MUT were co-transfected with mimic NC or miR-512-3p mimic into HUVECs using Lipofectamine 2000. At 48 h post-transfection, cells were lyzed and the luciferase activities were detected using the Dual-Luciferase Reporter Assay system (Promega, WI, USA).

### Statistical analysis

All experiments were performed in triplicates and data are presented as mean + standard deviation. One-way analysis of variance (ANOVA) followed by Tukey’s post hoc test was employed for statistical analysis among multiple groups. The statistically significant level was set to p < 0.05.

## Results

### Decreased proliferative and migrative activities and increased apoptosis of ox-LDL-treated HUVECs

**Figure 1.**
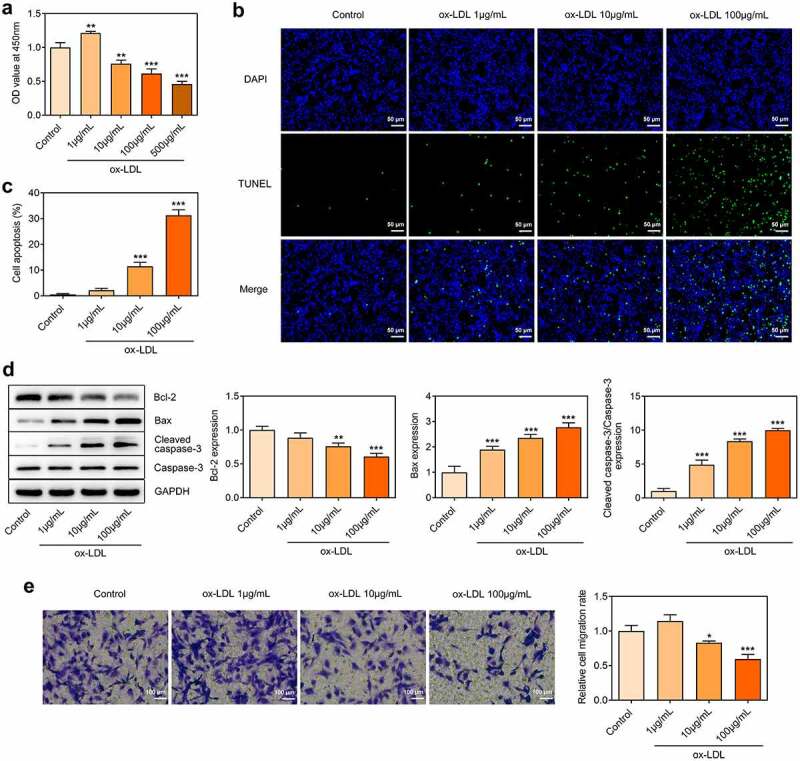
Decreased proliferative and migrative activities and increased apoptosis of ox-LDL-treated HUVECs. HUVECs were treated with 1, 10, 100, 500 µg/mL ox-LDL. (a) CCK-8 assay for determination of HUVECs viability. (b, c) TUNEL staining for determination of HUVECs apoptosis. (d) Western blot assay for determination of Bcl-2, Bax, Cleaved caspase-3 and Caspase-3 protein levels in HUVECs. (e) Transwell assay for determination of HUVECs migration. * p < 0.05, ** p < 0.01, *** p < 0.001 versus control

HUVECs were treated with 1, 10, 100, 500 µg/mL ox-LDL to establish the cell model of AS. Treatment with 1 µg/mL ox-LDL promoted HUVECs proliferation and treatment with 10, 100, 500 µg/mL ox-LDL inhibited HUVECs proliferation, both resulting in the injury of vascular endothelial cells (Figure 1(a)). Then, 1, 10, 100 µg/mL ox-LDL were chosen for subsequent experiments. Furthermore, increased TUNEL-positive cells suggested that treatment with 10, 100 µg/mL ox-LDL markedly boosted the apoptosis of HUVECs in a dose-dependent manner (Figure 1(b,c)). In addition, ox-LDL treatment reduced the level of anti-apoptotic protein Bcl-2 and elevated the levels of pro-apoptotic protein Bax and Cleaved caspase-3 (Figure 1(d)). Treatment with 1 µg/mL ox-LDL promoted HUVECs migration and treatment with 10, 100 µg/mL ox-LDL inhibited HUVECs migration (Figure 1(e)).

### Ox-LDL treatment dose-dependently induced autophagy in HUVECs

**Figure 2.**
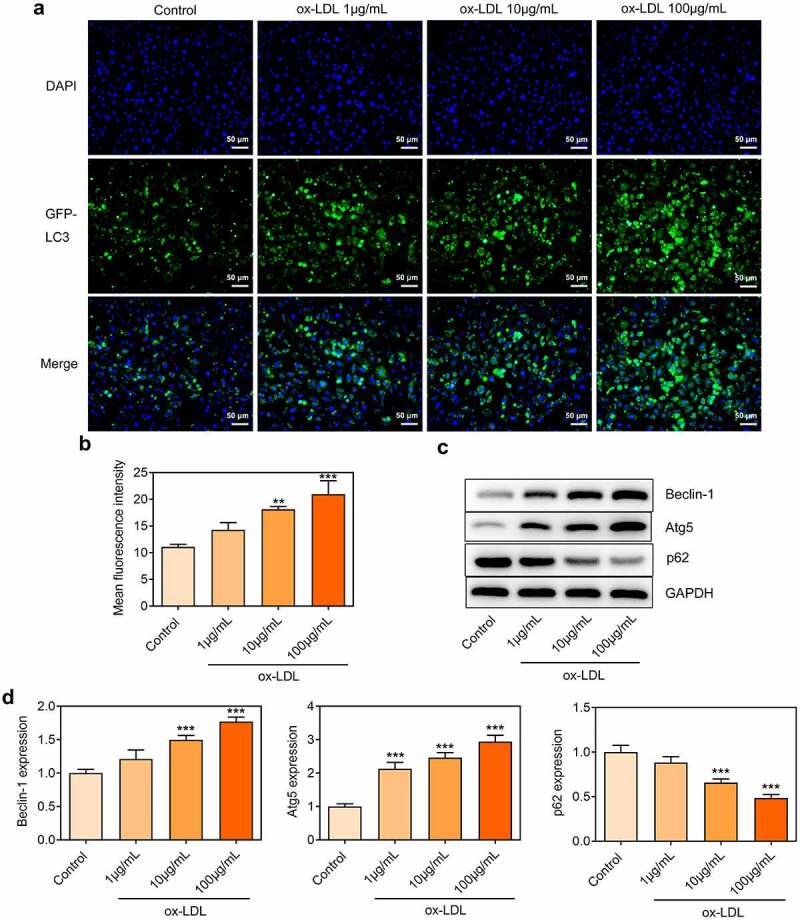
Ox-LDL treatment dose-dependently induced autophagy in HUVECs. HUVECs were treated with 1, 10, 100 µg/mL ox-LDL. (a, b) Immunofluorescence for determination of LC3 expression. (c, d) Western blot assay for determination of Beclin-1, Atg5 and p62 protein levels in HUVECs. ** p < 0.01, *** p < 0.001 versus control

LC3 is a component of the autophagosomal membranes. LC3 expression was detected through immunofluorescence assay. Punctate LC3 accumulation was dose-dependently increased in ox-LDL-treated HUVECs, which confirmed that ox-LDL treatment enhanced autophagy in HUVECs (Figure 2(a,b)). Western blot further revealed that ox-LDL treatment promoted the expressions of Beclin-1 and Atg5 and inhibited p62 expression (Figure 2(c,d)). Findings above suggested that ox-LDL treatment-induced autophagy in HUVECs in a dose-dependent manner.

### Ox-LDL treatment dose-dependently induced ER stress in HUVECs

**Figure 3.**
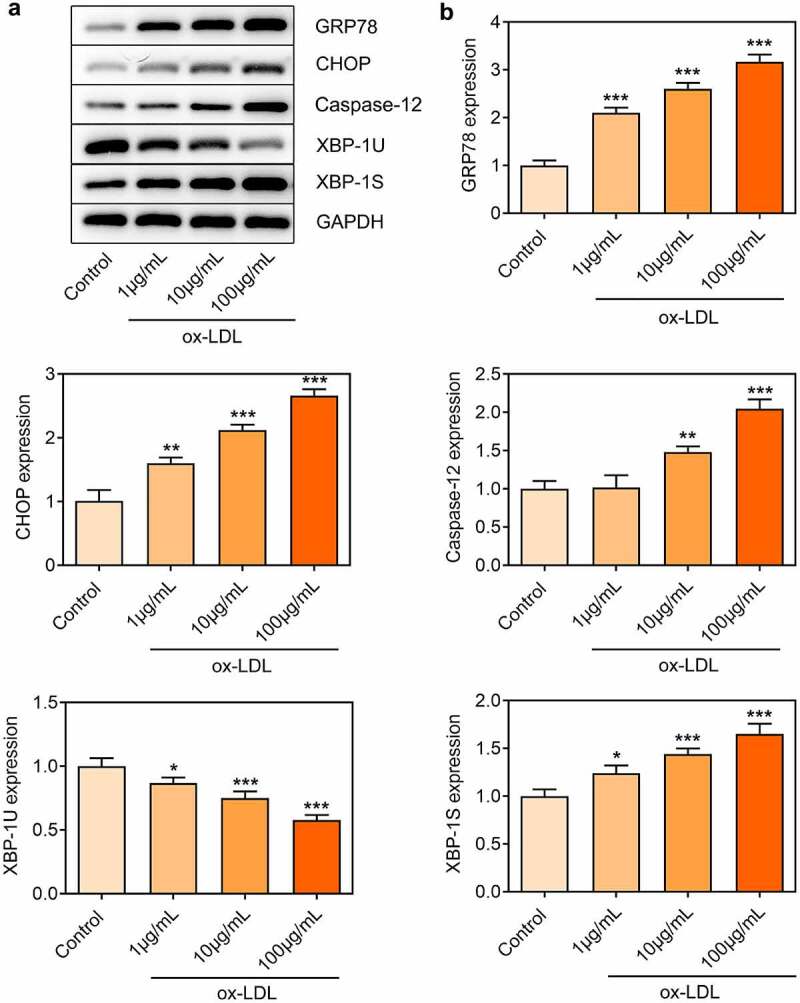
Ox-LDL treatment dose-dependently induced ER stress in HUVECs. HUVECs were treated with 1, 10, 100 µg/mL ox-LDL. (a, b) Western blot assay for determination of GRP78, CHOP, Caspase-12, XBP-1 U and XBP-1S protein levels in HUVECs. * p < 0.05, ** p < 0.01, *** p < 0.001 versus control

Considering the critical role of ER stress in the pathogenesis of AS, the expressions of ER stress-related proteins in ox-LDL-treated HUVECs were also investigated. Elevated expressions of GRP78, CHOP, Caspase-12 and XBP-1S and reduced XBP-1 U expression in ox-LDL-treated HUVECs were observed, which indicated that ox-LDL treatment enhanced ER stress in HUVECs (Figure 3(a,b)).

### Downregulation of miR-512-3p enhanced cell viability and suppressed cell apoptosis in ox-LDL-treated HUVECs

**Figure 4.**
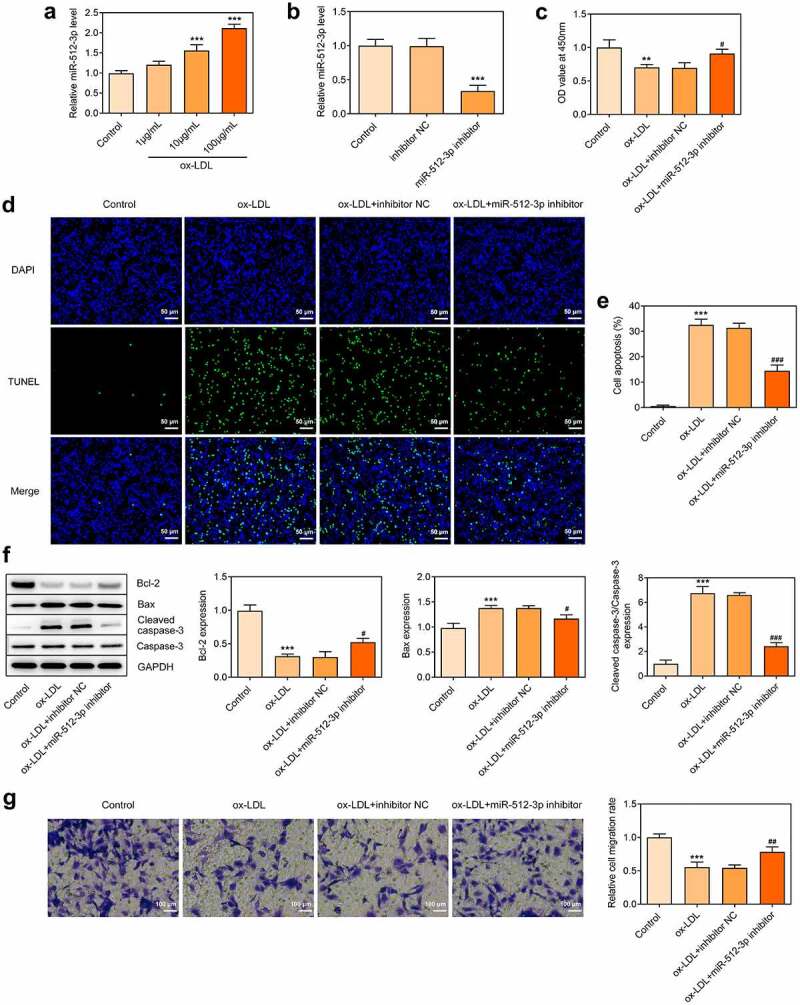
Downregulation of miR-512-3p enhanced cell viability and suppressed cell apoptosis in ox-LDL-treated HUVECs. (a) HUVECs were treated with 1, 10, 100 µg/mL ox-LDL. RT-qPCR for determination of miR-512-3p level in HUVECs. *** p < 0.001 versus Control. (b) HUVECs were transfected with miR-512-3p inhibitor or inhibitor NC. RT-qPCR for determination of miR-512-3p level in HUVECs. *** p < 0.001 versus inhibitor NC. (c) ox-LDL-treated HUVECs were transfected with miR-512-3p inhibitor or inhibitor NC. CCK-8 assay for determination of HUVECs viability. ** p < 0.01 versus Control, ^#^ p < 0.05 versus ox-LDL + inhibitor NC. (d, e) ox-LDL-treated HUVECs were transfected with miR-512-3p inhibitor or inhibitor NC. TUNEL staining for determination of HUVECs apoptosis. *** p < 0.001 versus control, ^###^ p < 0.001 versus ox-LDL + inhibitor NC. (f) ox-LDL-treated HUVECs were transfected with miR-512-3p inhibitor or inhibitor NC. Western blot assay for determination of Bcl-2, Bax, Cleaved caspase-3 and Caspase-3 protein levels in HUVECs. *** p < 0.001 versus Control, ^#^ p < 0.05, ^###^ p < 0.001 versus ox-LDL + inhibitor NC. (g) ox-LDL-treated HUVECs were transfected with miR-512-3p inhibitor or inhibitor NC. Transwell assay for determination of HUVECs migration. *** p < 0.001 versus Control, ^##^ p < 0.01 versus ox-LDL + inhibitor NC

ox-LDL treatment dose-dependently elevated miR-512-3p expression in HUVECs (Figure 4(a)). In order to determine the impact of miR-512-3p on AS, miR-512-3p inhibitor was transfected into HUVECs to downregulate miR-512-3p expression for functional experiments (Figure 4(b)). It was observed that downregulation of miR-512-3p enhanced the viability of ox-LDL-treated HUVECs (Figure 4(c)). Furthermore, reduced TUNEL-positive cells upon knockdown of miR-512-3p evidenced that downregulation of miR-512-3p suppressed the apoptosis of ox-LDL-treated HUVECs (Figure 4(d,e)). Downregulation of miR-512-3p promoted Bcl-2 expression and inhibited the expressions of Bax and Cleaved caspase-3, which also confirmed that knockdown of miR-512-3p could repress the apoptosis of ox-LDL-treated HUVECs (Figure 4(f)). Additionally, downregulation of miR-512-3p boosted HUVECs migration, partly abrogating the suppressing effect of ox-LDL treatment on HUVECs migration (Figure 4(g)).

### Downregulation of miR-512-3p attenuated ox-LDL-induced autophagy in HUVECs

**Figure 5.**
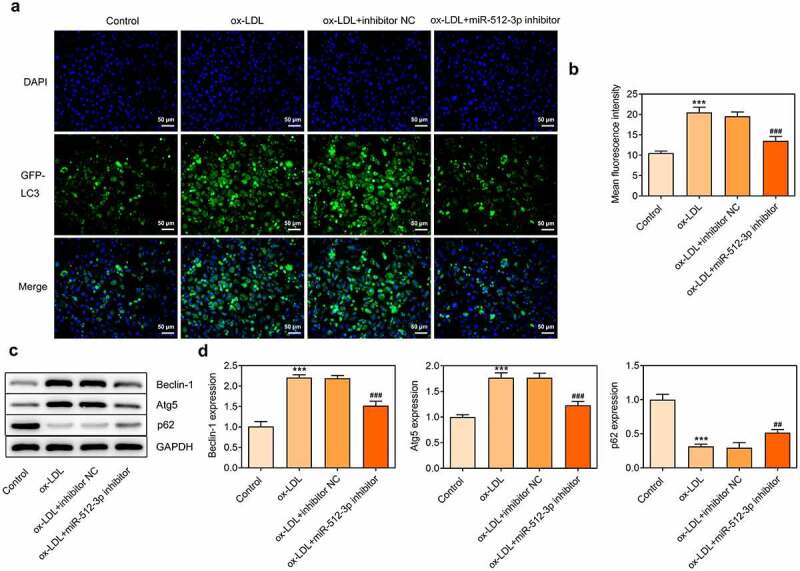
Downregulation of miR-512-3p attenuated ox-LDL-induced autophagy in HUVECs. ox-LDL-treated HUVECs were transfected with miR-512-3p inhibitor or inhibitor NC. (a, b) Immunofluorescence for determination of LC3 expression. (c, d) Western blot assay for determination of Beclin-1, Atg5 and p62 protein levels in HUVECs. *** p < 0.001 versus Control, ^##^ p < 0.01, ^###^ p < 0.001 versus ox-LDL + inhibitor NC

To further explore the in-depth function of miR-512-3p on ox-LDL-treated HUVECs, immunofluorescence for determination of LC3 expression and Western blot assay for detection of Beclin-1, Atg5 and p62 protein levels were performed to evaluate the autophagy level. Immunofluorescence results showed a significant decrease in LC3 (green dots) upon knockdown of miR-512-3p (Figure 5(a,b)). Furthermore, downregulation of miR-512-3p inhibited the expressions of Beclin-1 and Atg5 and promoted p62 expression in ox-LDL-treated HUVECs (Figure 5(c,d)). Based on these results, it can be ascertained that downregulation of miR-512-3p could alleviate ox-LDL-induced autophagy in HUVECs.

### Downregulation of miR-512-3p alleviated ox-LDL-induced ER stress in HUVECs by targeting XBP-1

**Figure 6.**
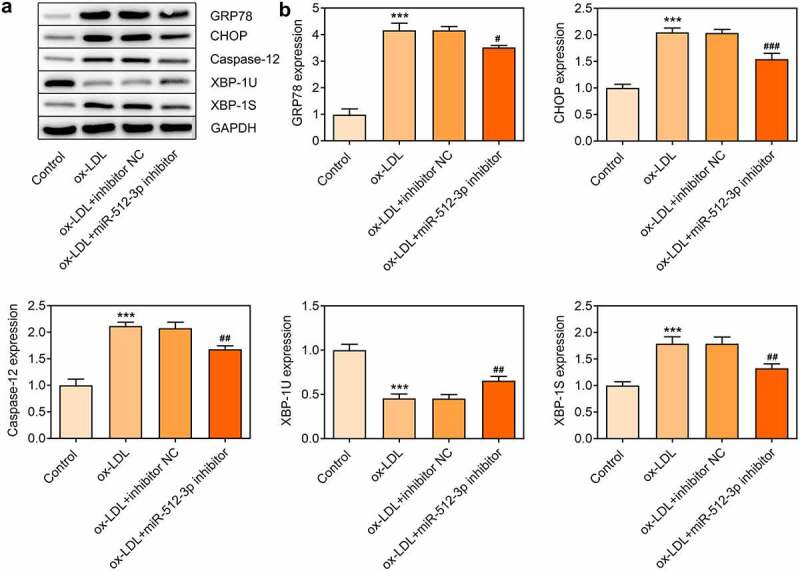
Downregulation of miR-512-3p alleviated ox-LDL-induced ER stress in HUVECs. ox-LDL-treated HUVECs were transfected with miR-512-3p inhibitor or inhibitor NC. (a, b) Western blot assay for determination of GRP78, CHOP, Caspase-12, XBP-1 U and XBP-1S protein levels in HUVECs. *** p < 0.001 versus Control, ^#^ p < 0.05, ^##^ p < 0.01, ^###^ p < 0.001 versus ox-LDL + inhibitor NC

**Figure 7.**
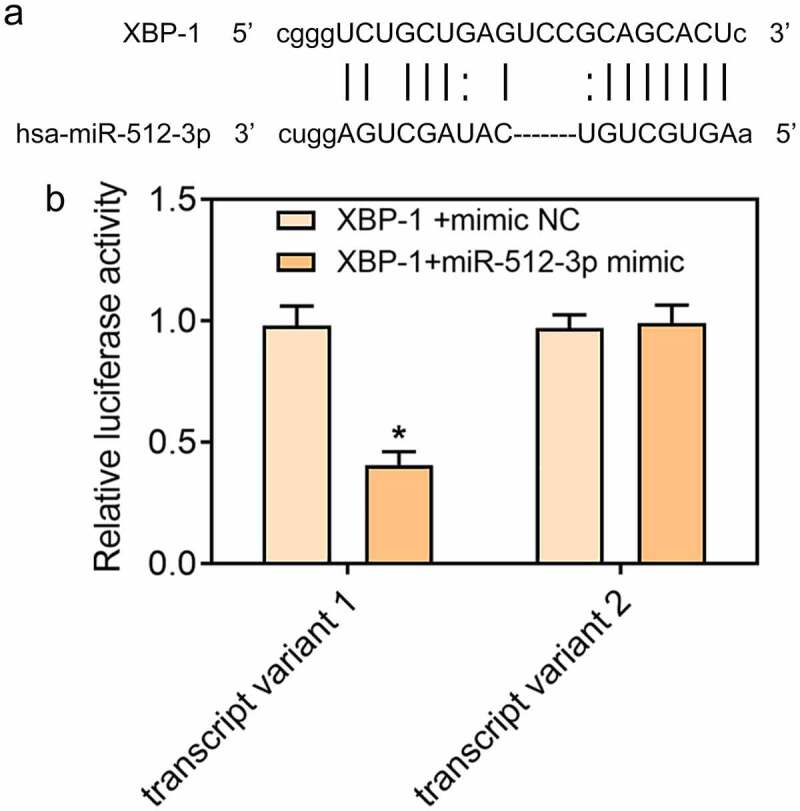
XBP-1 was a direct target of miR-512-3p. (a) starbase predicted the binding sites of XBP-1 to miR-512-3p. (b) Luciferase reporter assay for verifying the binding relationship between miR-512-3p and XBP-1. *p< 0.05 versus. XBP-1+ mimic NC

In ox-LDL-treated HUVECs, expressions of GRP78, CHOP, Caspase-12, and XBP-1S were decreased and XBP-1 U expression was increased upon knockdown of miR-512-3p, which confirmed that downregulation of miR-512-3p could alleviate ox-LDL-induced ER stress in HUVECs (Figure 6(a,b)). Additionally, starbase predicted the binding sites of XBP-1 to miR-512-3p (Figure 7(a)) and luciferase reporter assay verified the binding relationship between miR-512-3p and XBP-1 (Figure 7(b)). These data prompted that knockdown of miR-512-3p may attenuate ox-LDL-induced ER stress in HUVECs by targeting XBP-1 to manipulate the ratio of XBP-1S/XBP-1 U.

### Downregulation of miR-512-3p alleviated atherosclerotic lesions in AS model mice

**Figure 8.**
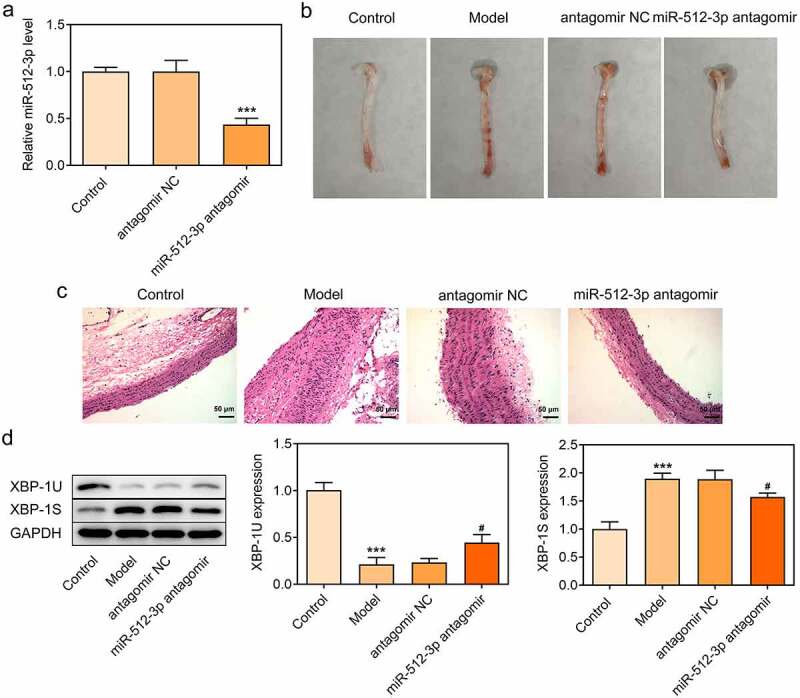
Downregulation of miR-512-3p alleviated atherosclerotic lesions in AS model mice. ApoE-/- mice were nourished with a high-fat diet to establish an in vivo model of AS. miR-512-3p antagomir or antagomir NC were injected into ApoE-/- mice through the tail vein. (a) RT-qPCR for determination of miR-512-3p level. *** p < 0.001 versus antagomir NC. (b) Photos of aortic tissues and atherosclerotic plaques. (c) H&E staining for evaluating atherosclerotic lesions of aortic tissues. (d) Western blot assay for determination of XBP-1 U and XBP-1S protein levels. *** p < 0.001 versus Control, ^#^ p < 0.05 versus antagomir NC

To further validate the biological functions of miR-512-3p on AS, ApoE-/- mice were nourished with a high-fat diet to establish an in vivo model of AS and miR-512-3p antagomir was injected into ApoE-/- mice through the tail vein to downregulate miR-512-3p expression (Figure 8(a)). In comparison with normal mice, obvious aortic atherosclerotic area was observed in AS model mice. Importantly, miR-512-3p antagomir greatly reduced aortic atherosclerotic area (Figure 8(b)). Moreover, it was observed that the carotid artery wall in the aortic vessels was thickened in AS model mice and the carotid artery wall was markedly thinned by miR-512-3p antagomir delivery (Figure 8(c)). In addition, decreased XBP-1 U expression and increased XBP-1S expression were observed in AS model mice, which were reversed by miR-512-3p antagomir delivery (Figure 8(d)).

## Discussion

AS is recognized as a vital pathological basis of cardiovascular disease [3]. Endothelial dysfunction caused by vascular endothelial cell injury has a certain relationship with the occurrence and development of AS [22].

Recent researches have identified that miRNAs are attracting more attention due to their key regulatory roles in the pathogenesis of AS [10,11]. For example, literature has reported that miR-590 could inhibit atherosclerotic lesion in the in vivo models of AS and facilitate the proliferation and suppress the apoptosis of ox-LDL-treated human aortic endothelial cells [23]. Additionally, it is demonstrated that miR-185 silencing could enhance the proliferative, migrative and invasive activities of ox-LDL-treated MOVAS cells [24]. Herein, it was demonstrated that ox-LDL treatment dose-dependently elevated miR-512-3p expression in HUVECs. Downregulation of miR-512-3p enhanced the viability, suppressed the apoptosis and promoted the migration of ox-LDL-treated HUVECs as well as alleviated atherosclerotic lesions in AS model mice.

Numerous studies suggest that ER stress plays an important role in the pathogenesis of various cardiovascular diseases. Hong et al. have demonstrated that ox-LDL could induce endothelial cell apoptosis through activation of ER stress pathway [25]. Hamczyk et al. [26] have indicated that progerin could accelerate AS progression by activating ER stress. In the present study, it was discovered that ox-LDL treatment induced the activation of ER stress in HUVECs, which was partially abolished upon downregulation of miR-512-3p. XBP-1 is a major transcriptional regulator of the UTR, which is responsible for regulating a subset of ER resident chaperone genes [27]. It was found that XBP-1 was a direct target of miR-512-3p. Downregulation of miR-512-3p decreased XBP-1S expression and increased XBP-1 U expression, indicating that miR-512-3p silencing attenuated ox-LDL-induced ER stress in HUVECs by targeting XBP-1 to manipulate the ratio of XBP-1S/XBP-1 U.

ER stress initiates the UPR and cells are induced apoptotic and autophagic death when UPR reaches a certain extent [28]. Autophagy is a widespread and unique degradation process in eukaryotic cells. When cells are under various stress conditions, autophagy will be initiated to maintain the stability of the internal environment and ensure normal proliferation and differentiation [7]. A lot of documents have verified that the development of AS is closely associated to autophagy. Zhang et al. state that downregulation of miR-155 could inhibit the autophagy of endothelial cells induced by ox-LDL [29]. In this current work, it was confirmed that miR-512-3p silencing attenuated autophagy in ox-LDL-treated HUVECs.

## Conclusion

To sum up, this work expounded the biological functions of miR-512-3p in ox-LDL-treated HUVECs and in vivo model of AS and investigated the underlying molecular mechanism. Downregulation of miR-512-3p enhanced the viability, suppressed the apoptosis, and promoted the migration of ox-LDL-treated HUVECs, alleviated atherosclerotic lesions in AS model mice as well as repressed autophagy and ER stress by targeting XBP-1 to manipulate the ratio of XBP-1S/XBP-1 U. Findings of this research prompted that miR-512-3p might serve as a therapeutic target for AS and may provide the theoretical foundation for therapies of atherosclerotic vascular diseases clinically.

## Data Availability

The analyzed data sets generated during the present study are available from the corresponding author on reasonable request.
